# Plant a Vine

**Published:** 1876-02

**Authors:** 


### PLANT A VINE.

We may safely calculate that ten thousand persons live in their own houses in New York city, and that in the rear of each of these houses are five hundred square feet of land, used as a back yard. Eight grape vines would flourish in this space, and each vine, of the "Isabella" variety, if well attended to, will produce two thousand bunches of delightful fruit, or sixteen thousand clusters. Downing says he has seen "one Isabella vine produce, in one season, three thousand fine clusters of well-ripened fruit." But suppose each vine yielded one hundred pounds only, the yield of eight vines would retail for one hundred and twenty dollars. But single grape vines have been known to yield one thousand pounds of grapes, and eighteen cents per pound is the retail price of good grapes in New York.

The want of employment is at the foundation of the ill health of multitudes of women and youth in large cities. We have frequent occasion to feel in reference to women who apply to us for medical advice, "*the best medicine for you is the wash-tub, at fifty cents a day.*"

Any growing family might appropriate a vine to its different members, with a rivalry as to whose vine would produce the largest crop. How many hours would thus be saved from idleness, from the street and its corrupting associations, from that wearing unrest or destructive listlessness which overtakes those girls and boys who have nothing to do, and are not obliged to do anything? The healthful advantages of this interested out-door employment cannot be readily estimated. Besides all this, the minds of the young would be naturally led to study the nature of the vine in particular, and of vegetation in general—the laws of vegetable life, the nature of manures, of the ingredients of the food for plants, the elements of vegetable nutrition—leading the mind off into mineralogy and geology, to say nothing of the flowering of plants, the blossoming of trees, and the various side studies into which these investigations would lead; thus giving a practical value and a felt interest in the studies of our schools, which boys and girls seldom experience. Who does not know that the theoretical study of botany, geology, and mineralogy in our schools is as dry to the scholars as the splinter of a fence-rail? Thus it is, and very naturally, that we seldom find a boarding-school girl, or one from a fashionable city institute, who knows anything at all of these deeply interesting sciences beyond the most general definitions. It is a disgrace and a shame to both parents and teachers, that our children, especially our daughters, so uniformly "*finish their studies*," and know nothing well. It is because nothing is done to make these studies practical, to lead the mind to take hold of them with interest and relish.

Proper attention to a single vine will employ a youth an hour a day, on an average, from May to November, with some little besides during the winter. And in the hope that some of our readers may be induced to heed our suggestions, we here give a history of what we practise ourselves.

Go at once, any time in April or early in May, to a horticultural nursery, and bespeak several *well-rooted vines*, not "cuttings;" thus you will save several years, as the vines will begin to bear the next season. Then prepare the ground, by throwing out the earth three feet deep and as much in diameter; throw in a foot and a half in depth of manure, consisting of bones, coffee grounds, egg shells, fish heads—in short, any of the offal of the kitchen, or chip manure, or old mortar; put on this half a peck of wood ashes, mixed up with as rich earth as you can get. The Concord, Diana, and Isabella varieties are best for this latitude, and it might be of interest to have different kinds. The gardener will set them in for you, charging you about a dollar and a half for a good vine and all. Lay around the vine, on the surface, some straw or leaves, in order to keep the earth moist by preventing rapid evaporation; over this throw the coffee and tea leavings, as also all the soapsuds of weekly and other washings, for the effect of soapsuds on the vine, as well as other plants, in giving them life and vigor, is surprising. Under such a treatment as this you will, in three or four years, not only have a liberal crop of delicious fruit, but a refreshing shade and a beautiful ornament to your dwellings.

After the first year, in November, the vines should be pruned—that is, when they have grown as large as you wish them to extend. To prevent their further growth, the young wood, the growth of the preceding summer, and which bore the grapes, should be cut off, within two or three buds of its union with the parent stem; it is known by its smoother and lighter-colored bark.

As soon as the grapes are formed, nip off all the shoots that appear afterwards, so as to throw all the strength of the vine into the producing ones; this must be done every two weeks during the summer.

Never pinch off the leaves to hasten the ripening of the fruit; nature has placed them there to facilitate that ripening in her own way.

The grapes are not really good until the first of October, and every day they become more delicious until the first week in November, when, in the middle of one of the driest and most sunshiny days of our delightful autumn, the remaining grapes should be picked and placed on wooden shelves in the cellar, suspended from the ceiling, and not within two feet of the wall or floor, or anything else by which a rat could reach them. On the shelf a layer of straw, two inches deep, should be placed; paper is not so good, but is safer from fire. Place the grapes on this straw, two bunches deep, with another layer of straw over them, and with ordinary precaution, with our New York cellars, they will keep until spring, growing sweeter every day. They shrivel up a little, but all the substance is there—the only drawback being a little mustiness, but not more, we think, than is perceived in the white grapes from abroad, packed in sawdust. Our own kept this last year until February, and would have kept longer had we not used them so liberally—that is, wifey and I, and a big rat, who by the aid of a water-pipe climbed up the cellar wall, and so feasted on the pure juice of the grape all winter, making a wine-press of his paws.

It requires more cold to freeze grapes on the vine, than to freeze water; so that those designed for current use may remain on the vines, to be picked from day to day, as needed, until the first of December. Nothing, perhaps, is gained by allowing the vines to spread greatly. In regular vineyards they are allowed to grow about seven feet high, to a stake, to which arms are attached projecting three feet on either side—the vines being seven feet apart; thus an acre takes a thousand plants, yielding an average profit for wine-making purposes of three hundred dollars a year per acre.

